# Book Reviews

**Published:** 1799-06

**Authors:** 


PRACTICE OF MEDICINE AND SURGERY.

*Medicina Nautica: an Effay on the Difeafes of Seamen: with an Appendix, containing Communications on the new Doctrine of Contagion and Yellow Fever, by American Phyficians; tranfmitted to the Admiralty by Sir John Temple, Bart., his Majefty's Conful General.* Vol. II. By THOMAS TROTTER, M. D. Phyfician to his Majefty's Fleet, &c. *London,* (Price 7s. 6d.) Longman and Rees.

We have already announced this volume, and its important contents, in our firft Number, p. 80.

In juftice to the author, we fhall firft give an account of its particular contents, and then quote a few paffages refpecting the new doctrine of preventing and deftroying contagion, by means of the nitrous vapour; on which fubject we refer the reader to Dr. J. C. Smith's work, noticed in our fecond Number, p. 213.

After a fhort 'Introduction,' which principally relates to the eftablifhment of a Medical Library, and the delivering of Clinical Lectures, at the Royal Hofpitals of Haflar and Plymouth, Dr. Trotter furnifhes us (from p. 11 to 31) with a "*General Abftract of the State of Health in the Fleet, for the years* 1797 *and* 1798."

In

In the subsequent chapters (from p. 32 to 266) the author treats of *Contagion; Yellow Fever; Small-pox; Epidemical Ophthalmia; on the Effect of Nitrous Acid in the Venereal Disease;—Miscellaneous Communications and Remarks; on Diet; the Malignant Ulcer; Experiments on the Nitrous Gas;* several cases of *Fractures;* one of large *Abscesses on the Thigh;* and one of *Locked jaw;* communicated by different navy-surgeons.

On the subject of destroying contagion, by means of fumigating ships and wards in hospitals with nitrous vapour, we extract the following severe animadversions:

"So absurd and inconsistent", says Dr. Trotter, "are prevailing opinions and habits of imitation, in particular affairs among mankind, that what is called a poison one day, may have the chance to be vended as an infallible remedy the day after. The sulphurated hydrogenous gas, when mixed with a proportion of carbonic acid gas, is no other than the aërial vapour produced from the explosion of combustion of gun-powder, which has been so long used in his Majesty's ships for the destruction of contagion. It affords an ample but lamentable proof of the wavering and imbecility of all human reasoning. Dr. Smith himself, we are very apt to believe, must have been strongly impressed with the dread of unfolding the weak sides of his doctrine, that he says nothing of the primitive principles of his nitrous gas. That must have soon reduced him to inexplicable difficulties; for he must have sought its constituent parts in the final decomposition of animal matter, by putrefaction; and after being evolved from his pipkins, whether in ships or hospitals, from its well-known elective attractions, he must have traced it through all its various degrees of oxygenation, till it became nitrous acid, without imparting a single quality to the air to support life; but, on the contrary, to render it so much the worse, by the quantity it required to bring it to the state of an acid. P. 45 and 46.

"After all the latitude which the advocates for fumigation have thus given to its properties for purifying the air, and preventing diseases, it can certainly never be employed in *healthy ships*, for any reason yet produced from experience, or from chemistry. In crowded situations, when the vital portion of the atmosphere is diminished by respiration, it can only be replenished by a fresh column from without, or by some process giving out oxygen. Nitrous gas, though possessing it in combination, cannot give it out under these circumstances; on the contrary, it tends still more to lessen the respirable portion, from its strong attraction for oxygen, which it greedily combines with to the point of saturation. This quality of nitrous gas has been so generally noticed by chemists, that it is employed every where as an eudiometer for measuring the purity of any given quantity of atmospheric air; and is, I believe, the ingenious invention of Dr. Priestley." P. 47.

A considerable part of this volume (from p. 276 to 471) is occupied with the '*Appendix*', which contains a summary of Dr. Mitchill's doctrine relative to *pestilential fluids*, as displayed in his '*Defence of the Soap and Candle-makers*', at New-York, in March, 1797; and in the three *Dissertations on Pestilential Fluids;* by Drs. Saltonstall, Bay, and Lent; the last of which we have noticed in our first Number, p. 98.—We regret that our limits will not admit of giving, at present, even a concise account of that ingenious *new doctrine*, but we shall endeavour to supply this deficiency on a future occasion; meanwhile the reader is referred to a summary of its principles contained in a paper entitled, '*Outlines of Medical Geography*', by Dr. Mitchill, inserted in our third Number, from page 254 to 259.

*Essays on the Venereal Disease, and its concomitant Affections:* By William Blair, *A. M.* Surgeon of the Lock Hospital and Finsbury Dispensary, &c. first part of the first Essay. A new Edition. corrected. 8vo. 252 pp. 1799. (Price 4s.) *London*, Johnson.

In the Preface to this edition the author says: "The uncommon succes of several surgeons in their late experience with the acid (especially at the Woolwich Hospital) has induced me to select a few well-marked examples of lues venerea, for the purpose of repeating my observations, and of discovering any error or defect there might possibly have been in my practice last year. But no alteration whatever has taken place in my opinion, in consequence of these renewed trials: I therefore still conclude that the advocates for the acid are greatly deceiving themselves, and that too little discrimination has been used in ascertaining the *real* merits of Mr. Scott's plan. As a general substitute for mercury in the treatment of syphilis, it is certainly inadmissible. Its efficacy may be most depended on, in cases where a venereal taint is least capable of proof."

Our limits will not permit us to give any number of the cases in detail, nor do we think it necessary; as the author's opinion, formed from the result of these, and many others which he proposes shortly to publish, is given above; and as our readers could only judge of the truth of that opinion by a perusal of the whole number. The cases that struck us as the most decisive among Mr. Blair's own trials, principally in the Lock Hospital, on the primary symptoms of the disease, are, Case 2. p. 126, and Case 12. p. 139. On the secondary symptoms are, Case 6. p. 177; C. 13. p. 189; C. 23. p. 208; C. 9. p. 240; and C. 11. p. 249.

*Practical Observations on the Diseases of the Army in Jamaica, as they occurred between the years* 1792 *and* 1797: *on the situation, climate, and Diseases of that island; and on the most probable means of lessening mortality among the troops, and among Europeans in tropical climates.* By William Lempriere, Apothecary to his Majesty's Forces. In two vols. 291 and 361 pages, 8vo. with eight folio tables. 1799. (Price 13s. boards.) *London*, Longman and Rees.

In a well-written preface, the author apologizes (indeed without necessity) "for attempting to simplify this branch of medical knowledge, by minutely stating, in a plain, easy language, blended with no more theory than what is absolutely necessary to throw light on his remarks, the result of his observations, during a period of eleven years, constantly devoted to his Majesty's service in warm climates: the last six of these have been spent in the West-Indies, where the author performed the duty of regimental-surgeon, and afterwards the more important office of superintending the military hospitals in Jamaica. When not called off by his military avocations, he also was engaged in a very extensive line of private practice in Spanish Town, at the time when the greatest sickness prevailed in Jamaica. From these opportunities he derived the observations which, with some diffidence, he now presents to the public eye."

This work appears to us well arranged, and replete with truly practical information. We regret that it is not consistent with our limitted plan, to give a more particular account of its contents; and shall therefore point out, as particularly deserving the reader's attention, the eighth chapter of the first volume, '*On the prevention of sickness and mortalily among his Majesty's troops stationed in the West Indies*;' and the eleventh chapter of the second volume, '*On the duties of the regimental surgeon, the attendance of the sick,*

*and the arrangement of hofpitals in Jamaica.*" On each of thefe topics, as far as relates to naval fervice, our correfpondent, Mr. Henderfon, has treated at fome length, in the three preceding numbers of our Journal, pp. 91—96; 137—143; and 236—238; as well as in the prefent number, from p. 340—346. Yet we do not mean to infinuate that thefe important fubjects are exhaufted by either of thefe ingenious writers, or that there is not ftill ample room for the obfervations of others.

*Effay on the Caufes, early Signs, and Prevention of Pulmonary Confumption; for the ufe of Parents and Preceptors.* By THOMAS BEDDOES, M. D. 8vo. 274 pp. (Price 5s.) 1799. *Briftol,* Cottle; *London,* Longman & Rees.

The author prefaces this 'Effay' with a *View of the Subject,* in which he obferves, that nearly one fourth of the deaths in the Britifh iflands is, by the bills of mortality, referred to confumption; an affertion which he endeavours to fupport by a table fhewing the mortality, regiftered under the heads of *decline* and *confumption,* in one of the parifhes at Briftol, where the population is about 10,000, from the year 1790 to the year 1796, being a term of feven years. According to this table, of 1511 perfons, 683 are faid to have died of confumption or decline: fo that *nearly one half* fell victims to that mercilefs difeafe.

In the next fection of the Work, the author delivers the '*Plan of the Effay,*' from which we quote the following characteriftic paffages:

"In fearch of facts applicable in the fequel to my fubject, I fhall firft engage in a brief inquiry concerning thofe countries and claffes that enjoy more or lefs of exemption from confumption. Could a doctrine of exemption be eftablifhed, it might furnifh fomething ufeful, by way of moral. If we could difcern the circumftances on which exemption depends, we fhould only have to adopt them as nearly as poffible into our own conduct. On the other hand, if it fhall appear that there are whole defcriptions of perfons more liable to the complaint, we may ftand a chance of collecting from their hiftory a leffon equally ufeful, concerning the habits to be avoided.

"I fhould, perhaps, have ftill longer delayed the publication of thefe papers, had I not fuppofed the lately afcertained means of cure (in fome circumftances at leaft of true confumption) likely to awaken curiofity to the whole fubject. The fituation of Europe has alfo its weight in urging me forward. Not only is *the night coming, when no man can work,* but I am apprehenfive, likewife, that *the tempeft is gathering, which may fweep away the workman together with his work.*"

As we have already pointed out (p. 384 of the prefent Journal) the practical tendency of this volume, as far as relates to profeffional men, we fhall at prefent only call the attention of the reader to its multifarious contents, viz. On *Climate—Weft Indies; Portugal; Italy; Madeira.—Claffes exempt: Butchers; Fifh-wives; Sailors; Watermen.—Perfons more liable to Phthifis—Animals confumptive—(Of the Dutch)—Scotland—General Inference—Particular Confiderations—Of the Phthifical Exterior—The Blood-warm Bath—Bed-warmth—Cold Bath—Cold Air—Cool Bath—Approach of Confumption—Cure of Confumption.*

*A Detection of the fallacy of Dr. Hull's Defence of the Cæfarean Operation.* By W. SIMMONS, Member of the Corporation of Surgeons in London, and Senior Surgeon to the Manchefter Infirmary. 8vo. 103 pp. *Manchefter,* Clarkes; *London,* Vernor and Hood; *Edinburgh,* Creech.

In our preceding Number, p. 308, & feq. we have given a fhort account of the author's late publication intitled, "*Reflections on the propriety of performing*

*forming the Cæsarean Operation*," which, we are sorry to find, has given rise to the controversy now carried on between Mr. Simmons and Dr. Hull. We lament that this controversy is not conducted with that temper and decorum, which ought ever to accompany scientific inquiries.

*An Account of the Plague which raged at Moscow in* 1771. By CHARLES DE MERTENS, M.D. Translated from the French, with Notes. 8vo. 122 pp. 1799.

This treatise was originally written in Latin; a French translation of it was published in 1784, at Vienna; and the present translator informs us in the preface, that he has been induced to submit it to the attention of medical practitioners in this country, on account of the danger to which we are exposed, of importing the pestilential contagion from America, or from Turkey and the Levant. "No one," says he, "who is acquainted with the nature of that contagion, can deny the possibility of its importation from America into this country, either now or hereafter, by infected persons or infected merchandise. On the other hand, are we not threatened with a similar danger from the East? In executing the hostile opearations, which are going forwards in the Mediterranean, it seems scarcely possible for our fleets and armies to keep clear of contagion. No nation was ever long engaged in a war with the Turks without taking the plague. In this respect they are as much to be dreaded by their friends as their foes. If, in the present contest, Italy, and France, and England shall escape this scourge, it will form an exception to past events, which all Europe must devoutly pray for."

*Traité des maladies des voies Urinaires, &c. A Treatise on the diseases of the Urinary passages;* By P. J. DESAULT; extracted from the Chirurgical Journal, enlarged and published by XAV. BICHAT, 8vo. *Paris.* The widow Dessault, No. 18, *Cloitre, Notre Dame*, and Nicole, *Rue du Bouloy*.

This work is divided into two parts: the first treats of all the diseases connected with the urinary secretion—the second, of those relative to its excretion. The first part is divided into four chapters, in which diabetes, the suppression of urine, its corruption, and the urinary calculus, are successively treated of. Jos. Frank has taken notice (*Ratio institut clinici &c.*) of two species of diabetes, similar to those described by Dr. Rollo, the one insipid, the other saccharine. According to Dr. Frank, the causes of diabetes have a debilitating effect, directly and indirecty, and exhaust the vital principle, if we may judge from the cases of debauchees and libertines who are subject to that disorder. The celebrated Trenka, in his commentary on the diabetes thinks that it is the effect of passion and terror. Lastly, Cullen supposes, that it proceeds from a want of assimilation, which is also the opinion of La Place, laid down in his work "*De vera diabetis causa in defectu assimilationis quærendâ*, Goettingen, 1784. From all these opinions, Dr. Frank, suspects that diabetes proceeds from a local affection accompanied with general debility, and prefers in the cure of it a stimulent treatment.

